# Angle-Resolved Plasmonic Properties of Single Gold Nanorod Dimers

**DOI:** 10.1007/s40820-014-0011-7

**Published:** 2014-09-26

**Authors:** Jian Wu, Xuxing Lu, Qiannan Zhu, Junwei Zhao, Qishun Shen, Li Zhan, Weihai Ni

**Affiliations:** 1 Department of Physics and Astronomy, Key Laboratory for Laser Plasmas (Ministry of Education), State Key Lab of Advanced Optical Communication Systems and Networks, Shanghai Jiao Tong University, Shanghai, 200240 People’s Republic of China; 2 Division of i-Lab, Key Laboratory for Nano-Bio Interface Research & Collaborative Innovation Center of Suzhou Nano Science and Technology, Suzhou Institute of Nano-Tech & Nano-Bionics, Chinese Academy of Sciences, Suzhou, 215123 Jiangsu People’s Republic of China

**Keywords:** Plasmonics, Gold nanorods, Self-assembly

## Abstract

Through wet-chemical assembly methods, gold nanorods were placed close to each other and formed a dimer with a gap distance ~1 nm, and hence degenerated plasmonic dipole modes of individual nanorods coupled together to produce hybridized bonding and antibonding resonance modes. Previous studies using a condenser for illumination result in averaged signals over all excitation angles. By exciting an individual dimer obliquely at different angles, we demonstrate that these two new resonance modes are highly tunable and sensitive to the angle between the excitation polarization and the dimer orientation, which follows cos^2^*φ* dependence. Moreover, for dimer structures with various structure angles, the resonance wavelengths as well as the refractive index sensitivities were found independent of the structure angle. Calculated angle-resolved plasmonic properties are in good agreement with the measurements. The assembled nanostructures investigated here are important for fundamental researches as well as potential applications when they are used as building blocks in plasmon-based optical and optoelectronic devices.

**Electronic supplementary material:**

The online version of this article (doi:10.1007/s40820-014-0011-7) contains supplementary material, which is available to authorized users.

## Introduction

In recent years, noble metal nanoparticles and their assembled nanostructures have attracted a great amount of attention because they show prominent and versatile optical properties in the visible range due to localized surface plasmon resonance (LSPR). In the assembled nanostructures, metal nanoparticles are placed close to each other, and the localized surface plasmon of individual nanoparticles can be coupled together giving rise to so-called “hot spots”, which are important for fundamental researches as well as potential applications in optics [1–4], chemistry [5–7], or electronics [8].

To date, studies based on calculations [8–11] and experiments [12–16] have been performed on the plasmonic coupling in metal nanoparticle-assembled dimer structures. Created by wet-chemical assembly methods, the dimer structures have much smaller gap distance than what can typically be achieved using electron-beam lithography fabrication methods (>5 nm) [17]. For a typical nanorod dimer structure with small gap distance, degenerate plasmonic dipole modes of individual nanorods will couple together to produce two new plasmonic resonances, antibonding and bonding modes respectively at higher and lower energies, whose separation is approximated by the Simpson-Peterson equation [9]. The symmetry breaking in the dimer structures induces the appearance of the normally dark antibonding modes as well as the dark-to-bright mode conversion [18, 19]. The two new resonance modes are highly tunable and sensitive to polarization orientation, which enables individual dimers to act as efficient orientation sensors [1, 20, 21]. However, these studies on individual nanorods are usually performed using a condenser for illumination with high numerical apertures where the signal obtained is averaged over all excitation angles. Little effort was made to study the effects of the polarization of excitation with respect to the dimer orientation, and therefore contribution from individual resonance modes cannot be discriminated.

In this paper, we performed a systematic study on the angle-resolved plasmonic properties of single gold nanorod dimer structures by exciting the dimer obliquely at specific or various angles. Gold nanorod dimer structures are created using cysteine (CYS) as linking molecules in aqueous solutions. V-shaped dimer structures are formed by depositing the assemblies from solutions onto a substrate. Strong plasmonic coupling occurs in these dimer structures, which results in the hybridized bonding and antibonding resonance modes. Single-particle dark-field scattering experiments indicate that the modes both are highly sensitive to the angle *φ* between the excitation polarization and the orientation of the dimer. The scattering intensity follows a typical cos^2^*φ* dependence due to the decomposition of the excitation electric field vector along either the bisector of the dimer for the radiative antibonding mode or its orthogonal direction for the radiative bonding mode. Effect of the angle between the two nanorods in the dimer on the scattering is also investigated by combining scanning electron microscope (SEM) and single-particle scattering spectroscopy. Both analytical and numerical methods are used in the calculations. The resonance wavelengths as well as the refractive index sensitivities are found independent of the structure angle. The calculated results are in good agreement with the measurements. Knowledge gained is helpful for the design of more complicated plasmonic structures, such as Fano and chiral structures. It is of particular interest for applications because these dimer structures can be used as building blocks in plasmon-based optical and optoelectronic devices.

## Results and Discussion

**Fig. 1:**
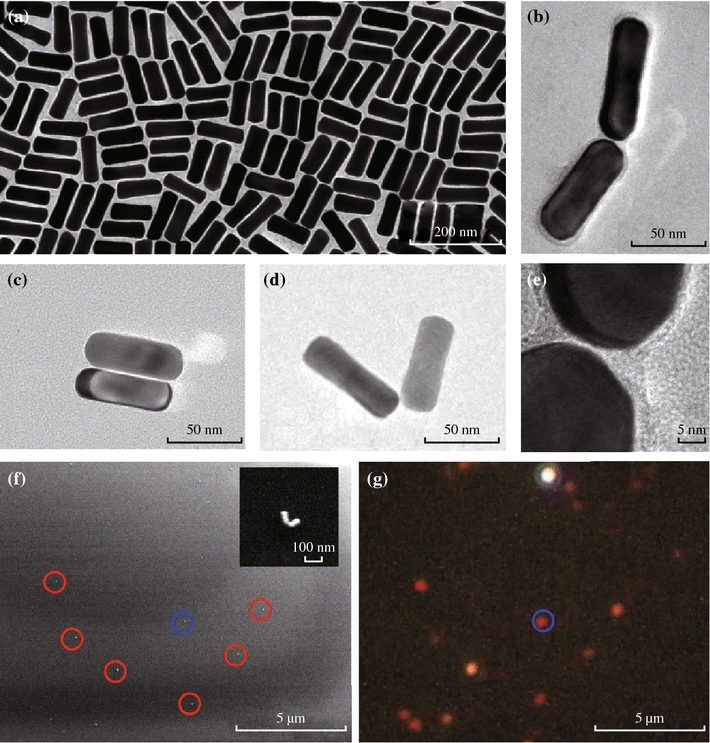
SEM and optical characterizations of gold nanorod assemblies. **a** TEM image of gold naonrods (69.3 ± 4.9 × 23.6 ± 1.8 nm, with an aspect ratio of 2.9 ± 0.3). **b**–**d** Representative TEM images of three assembled gold nanorod dimers. The angles between the two nanorods are 0°, 70°, and 149°, respectively. **e** High-resolution TEM image for the gap of the gold nanorod dimer in (**b**), indicating the gap distance between the two nanorods as about 1 nm. **f** SEM image for the gold nanorod dimers deposited on a cover glass substrate. The *inset* shows a SEM image for the dimer highlighted in the *blue circle* at high magnification. **g** Dark-field scattering image of the same area shown in SEM image (**f**). (Color figure online)

Gold nanorods were prepared using the seed-mediated growth method, and they are stabilized with cetyltrimethylammonium bromide (CTAB) in aqueous solutions. Figure 1a shows a transmission electron microscope (TEM) image of the as-synthesized gold nanorods. The average diameter, length, and aspect ratio of the nanorods are 23.6 ± 1.8 nm, 69.3 ± 4.9 nm, and 2.9 ± 0.3 nm, respectively. The gold nanorods show a longitudinal surface plasmon resonance at 717 nm in aqueous solutions (Fig. S1). Some of the gold nanorods show a slightly dog-bone shape. Compared to gold nanorods with ideal shape, the dog-bone nanorods possess a slightly red-shifted resonance wavelength [22]. The assembly of nanorods was performed by adding CYS to the solution as linking molecules, which is recorded spectroscopically (Fig. S1). Figures 1b–d show TEM images of representative dimer structures where two nanorods are connected at their ends through the linking molecules. The gap distance between the two nanorods was estimated to be about 1 nm according to the high-resolution TEM image (Fig. 1e). The gap distance between the two adjacent nanorods is largely determined by the linking molecule (CYS) and influenced by the CTAB surfactant bilayer. Based on the 3D construction of CYS molecule, the length starting from the thiol group and ending with the carboxyl group is approximately 0.6 nm. Because the molecular link involves two CYS molecules in an end-to-end fashion [23], gap distance is estimated to be 1.2 nm. This value is consistent with both TEM measurements and literature reports [4, 24, 25]. The assembled nanorods were then deposited from the solution onto a glass substrate. Figure 1f shows a SEM image of the nanorod dimers on the substrate. As shown in the inset, a dimer structure (highlighted in the blue circle) was identified with an angle *θ* = 80° between the long axes of the two nanorods. The length and diameter of the two nanorods were measured to be *l*_l_ = 71 nm and *d*_l_ = 24 nm (on the left), and *l*_r_ = 68 nm and *d*_r_ = 23 nm (on the right). The gap distance can hardly be measured under SEM because excess electron dose may damage the structure when SEM is working under high-magnification modes.

The dark-field scattering image and spectra of the individual gold nanorod dimers were measured using an Olympus BX53 optical microscope integrated with an Acton SpectraPro SP2750 monochromator and a Princeton Instruments PyLon 400BR charge-coupled device (CCD), which was cooled by liquid nitrogen to ~120 °C. The dimer structures deposited on glass slides were illuminated by white light from a 100 W Halogen lamp. The scattered light was collected with a long working distance 50X objective (N.A. = 0.5) and reflected to the entrance slit of the monochromator for imaging and spectroscopic measurements. Scattering spectra from individual dimers were calibrated by subtracting background spectra taken from the adjacent regions containing no nanostructures [26]. Single-particle measurements were performed by correlating the SEM and optical images. At the same area of the SEM image (Fig. 1f), a dark-field scattering image was taken for the gold nanostructures and shown in Fig. 1g. The diffraction-limited bright spots represent individual gold nanostructures and can be directly correlated with the nanostructures identified on the SEM image.

**Fig. 2:**
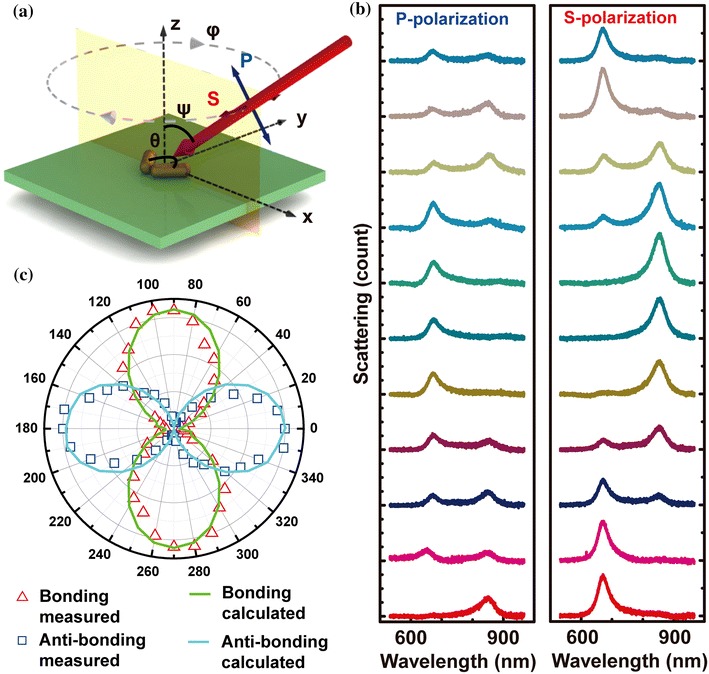
Angle-resolved plasmonic properties of an individual dimer. **a** Schematic design of the experimental setup for the angle-resolved measurements. A dimer with structure angle of 80*°* is investigated. The dimer is excited obliquely by an incident light at an incident angle *ψ*. The substrate can be rotated clockwise around the z-axis by an angle *φ*. Incident light with P- (*blue double-headed arrow*) and S-polarizations (*red double-headed arrow*) is indicated as the excitation source for the scattering measurements. **b** Scattering spectra from the dimer structure (*inset*, Fig. 1f) recorded respectively under the excitation of S- and P-polarizations. From bottom to top: scattering spectra with *φ* = 0°, 20°, 40°, 60°, 80°, 90°, 100°, 120°, 140°, 160°, and 180*°*. **c** S-polarization peak intensity of the two resonance peaks plotted as a function of *φ*. Measured peak intensities (*squares* and *triangles* are for peak intensities at 672 and 853 nm, respectively.) are compared with calculation. (Color figure online)

Figure 2a shows the schematic design of the experimental setup for the angle-resolved measurements. *θ* is defined as the angle between the long axes of the two nanorods in the dimer. The dimer is excited by an incident light at an incident angle *ψ*. The substrate can be rotated clockwise around the z-axis by an angle *φ*. Incident light with P- (blue double-headed arrow) and S-polarizations (red double-headed arrow) is indicated as the excitation source for the scattering measurements. Under the excitation of the incident light with P- and S-polarization, the scattering spectra of a dimer with *θ* = 80° was measured at *ψ* = 60°. By continuously rotating the substrate starting from *φ* = 0° to 180° at a step of 10°, scattering spectra from the same dimer structure were respectively recorded under the excitation of S- and P-polarizations, which is partially presented in Fig. 2b from bottom to top. Two peaks can be clearly identified at 672 nm and 853 nm in the spectra. With the increase of *φ*, as we can see for the S-polarization, the peak at 672 nm reaches a maximum when the polarization of excitation is parallel to the bisector of the nanorod dimer (*φ* = 0*°*), and decreases to zero when it is perpendicular to the bisector (*φ* = 90*°*). The peak at 853 nm just shows an opposite behavior. This periodic evolution can be clearly observed in Fig. 2c, where the peak intensity of the two resonance peaks is plotted as a function of *φ*. The behaviors of the two resonances show 90° difference in the angle dependence, suggesting that the orientations of these two resonance modes are orthogonal to each other.

**Fig. 3:**
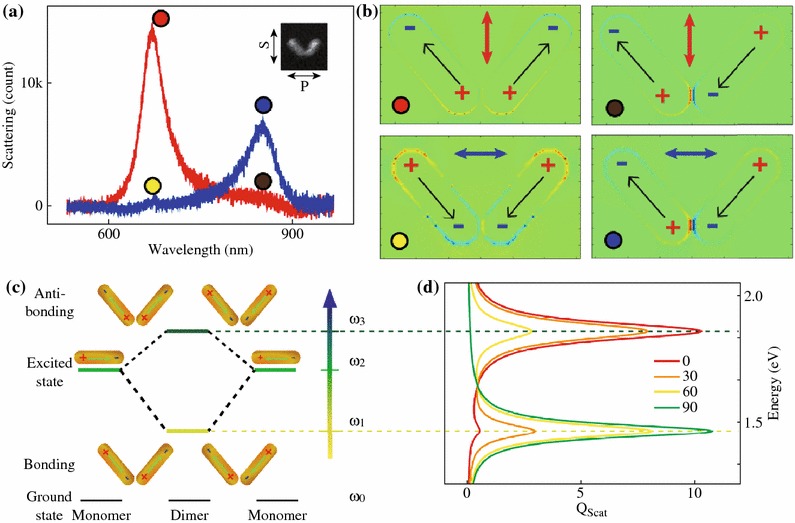
Plasmonic hybridization of the gold nanorod dimer. **a** A typical scattering spectrum obtained with specific excitation polarization. **b** Charge distribution profiles obtained using FDTD method. The *colors* indicate the relative sign of the charges. *Upper-left* S-polarization at 672 nm. *Upper-right* S-polarization at 853 nm. *Lower-left* P-polarization at 672 nm. *Lower-right* P-polarization at 853 nm. **c** Plasmonic hybridization theory scheme depicting the analogous hybridized excited energy. **d** Calculated scattering coefficient is plotted as a function of energy at various *φ* = 0°, 30°, 60°, and 90°. (Color figure online)

In order to understand the observed angle dependence of the plasmonic properties, both numerical and analytical methods are used for the theoretical simulations. For a typical scattering spectrum obtained with specific excitation polarization (Fig. 3a), the scattering peaks can be assigned to plasmonic resonance modes through calculating charge distribution profiles at the resonance wavelengths. Figure 3b shows the charge distribution profiles obtained using finite difference time domain (FDTD) method [27, 28]. Under the excitation of light with S-polarization, the individual dipoles oscillate symmetrically at 672 nm and produce a resultant dipoles along the bisector of the dimer (Fig. 3b upper-left), which give rise to the corresponding resonance scattering peak in the spectrum (red circle). On the contrary, at 853 nm, they oscillate anti-symmetrically and tend to cancel each other (Fig. 3b upper-right), which give rise to a zero resultant dipole and dark in scattering (brawn circle). As for the *P*-polarization, symmetric and anti-symmetric oscillations of the individual dipoles (Fig. 3b lower-left and lower-right) result in dark (yellow circle) and bright in scattering (blue circle), respectively. To get deeper insights into the physics behind plasmonic properties of the gold nanorod dimer, we conceive a simple plasmonic hybridization picture for the description of the electromagnetic behavior of the gold nanorod dimer. As shown in Fig. 3c, when the two gold nanorods are placed close to each other, due to strong plasmonic coupling the degenerate plasmonic resonance of individual gold nanorods is split into two hybridized resonance modes at lower and higher energies, corresponding to bonding and antibonding plasmonic modes, as well as anti-symmetric and symmetric collective charge density oscillations, respectively [15, 29]. The excitation efficiencies of these two hybridized modes are highly dependent on the relative orientation between the dimer structure and the polarization of the excitation light. At an arbitrary angle *φ*, the excitation light with specific polarization can be decomposed into two parts. One is along the bisector of the nanorod dimer which is related to the excitation of the radiative antibonding mode and the other is orthogonal and related to the radiative bonding mode. The fraction of the decomposition actually determines the relative height of the scattering at the two resonance energies.

Coupled dipole approximation (CDA) method was employed to investigate the angle dependence of the plasmonic properties of the gold nanorod dimer. Each gold nanorod is approximated as a prolate ellipsoid with a polarization tensor ${\overset{↔}{\alpha }}_{j}=T_{j}^{-1}{\overset{↔}{\alpha }}_{0}T_{j}$ (*j* = 1, 2), where ${\overset{↔}{\alpha }}_{0}$ is diagonal matrix with one longitudinal element *α*_L_ and two transverse elements *α*_T_ in the principal axes system of the rods, and *T*_*j*_ is the rotation matrix which relates the ellipsoid frame with the lab frame. By using Rayleigh-Gans approximation, the polarization elements can be given as [30, 31]:

$$\alpha_{j}=\frac{V}{4\pi }\frac{\varepsilon -\varepsilon_{m}}{\varepsilon_{m}+\left(\varepsilon -\varepsilon_{m}\right)L_{j}},$$

where *V* is the volume of the nanoparticle, *ε*_*m*_ is the dielectric constant of the surrounding medium, and *ε* is the dielectric function of gold [32].

The depolarization factors *L*_*j*_ are defined as

$$L_{L}=\frac{1-e^{2}}{e^{2}}\left[\frac{1}{2e}\ln\left(\frac{1+e}{1-e}\right)-1\right]$$

$$L_{T}=\frac{1-L_{L}}{2}$$

where the eccentricity *e* is defined as $e=\sqrt{1-b^{2}\;/\;\;a^{2}}$, and *a* and *b* are the major and minor radii of the ellipsoid, respectively [30, 31]. The polarization of each ellipsoid can be expressed as [33]:

$$p_{j}={\overset{↔}{\alpha }}_{j}\cdot \left(E_{\text{inc},j}+{\overset{↔}{G}}_{j,i}\cdot p_{i}\right)$$

where $E_{\text{inc},i}=E_{0}e_{\left(s,p\right)}\exp\left(ik\cdot r_{i}-i\omega t\right)$ is the electric field at r_i_ due to the incident plane wave. The subscript label S and P denote two orthogonal polarized lights with s- and p- polarization, respectively. The unit vectors of the s-polarization *e*_*s*_ (perpendicular to the incident plane), p-polarization *e*_*p*_ (parallel to the incident plane), and the wave vector *e*_*k*_ were chosen to obey right-handed rule $e_{k}=e_{s}\times e_{p}$. ${\overset{↔}{G}}_{j,i}\cdot p_{i}$ is the electric field due to the dipole at position r_i_ (*i* ≠ *j*) [33]:

$${\overset{↔}{G}}_{j,i}\cdot p_{i}=\frac{\exp(ikr_{ji})}{r_{ji}^{3}}\left\{\frac{\left(1-ikr_{ji}\right)}{r_{ji}^{2}}\times \left[3r_{ji}\left(r_{ji}\cdot p_{i}\right)-r_{ji}^{2}p_{i}\right]-k^{2}r_{ji}\times \left(r_{ji}\times p_{i}\right)\right\}$$

where $r_{ji}=r_{j}-r_{i}$, $r_{ji}=\left|r_{j}-r_{i}\right|$

Therefore, coupled equation for the dipole P_j_ (*j* = 1, 2) can be written as

$$\sum_{i=1}^{2}\left(\delta_{j,i}\overset{↔}{I}-\overset{↔}{\alpha }\cdot {\overset{↔}{G}}_{j,i}\right)\cdot p_{i}=\overset{↔}{\alpha }\cdot E_{\text{inc},j}$$

By solving these equations for the unknown polarizations P_j_, the extinction and absorption cross section can be calculated as:

$$C_{ext}=\frac{4\pi k}{{\left|E_{\text{inc}}\right|}^{2}}\sum_{j=1}^{2}\text{Im}\left(E_{\text{inc},j}^{*}\cdot p_{j}\right)$$

$$C_{abs}=\frac{4\pi k}{{\left|E_{\text{inc}}\right|}^{2}}\sum_{j=1}^{2}\left\{\text{Im}\left[p_{j}\cdot {\left(\alpha_{j}^{-1}\right)}^{*}\cdot p_{j}^{*}\right]-\frac{2}{3}k^{3}p_{j}\cdot p_{j}^{*}\right\}$$

with the scattering cross-section *C*_*sca*_ = *C*_*ext*_−*C*_*abs*_.

The scattering coefficient is calculated using the CDA method and plotted as a function of energy at various *φ* in Fig. 3d. As shown in the figure, the relative height of the scattering peaks at two resonance modes continuously change when *φ* is increased. When the calculated angle dependence of the scattering intensity is compared with the measurement, a satisfactory agreement is found (Fig. 2c). Note that the intensity follows a typical cos^2^*φ* dependence [4, 15, 34]. This is because the excitation *E*-field vector can be decomposed along either the bisector of the dimer for the radiative antibonding mode or its orthogonal direction for the radiative bonding mode. Through straight forward triangular calculation, one can find the excitation of antibonding mode have a factor of cos*φ*. In terms of intensity, the square of the *E*-field, the dependence of scattering intensity should then take a factor of cos^2^*φ*.

**Fig. 4:**
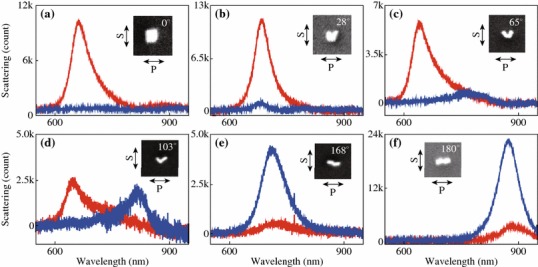
Scattering spectra from nanorod dimer structures with various *θ*. From **a** to **f**, the structure angles are 0°, 28°, 65°, 103°, 168°, and 180°, respectively. Zoomed-in SEM images of the dimer structures are shown in the *inset*. S- (*red*) and P-polarization (*blue*) are respectively parallel and perpendicular to the bisector of the nanorods. (Color figure online)

**Fig. 5:**
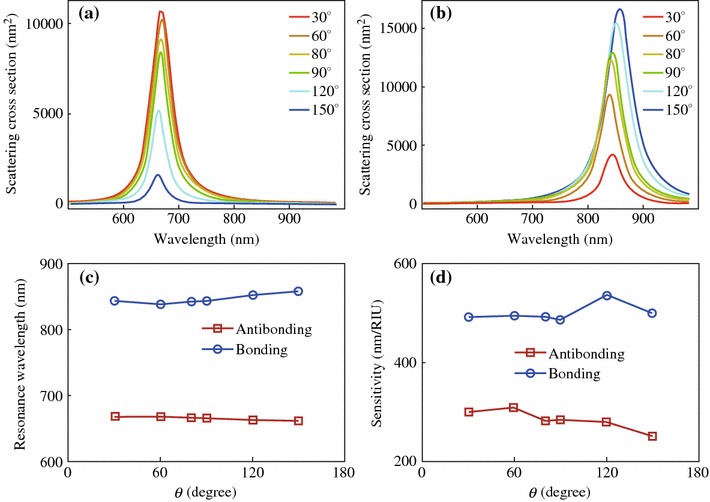
Plasmonic properties of the dimer structures with various structure angle *θ*. **a** Calculated scattering cross sections of the dimer under the excitation of light with S-polarization. **b** That for P-polarization. **c** Peak wavelengths of the two resonance modes as a function of structure angle. **d** Refractive index sensitivity as a function of structure angle

We next investigate the plasmonic properties that are dependent on the structure angle *θ*, the angle between the long axes of the two individual nanorods in the dimer. Figure 4 shows the scattering spectra from nanorod dimer structures with various *θ*. By varying *θ* in the range from 0° to 180°, the dimer structures exhibit antibonding and bonding resonance scattering peaks at about 650 and 850 nm, respectively. This result is consistent with the previous findings that the two resonance peaks are almost fixed at certain wavelengths [4, 18, 19]. As for the peak intensity, the two polarization states show opposite dependence. In the case of S-polarization, the intensity of the antibonding resonance scattering peak decreases with the increase of *θ*, and diminishes when *θ* = 180*°*. On the contrary, that of the bonding resonance peak increases with the increase of *θ*. In order to explain these experimental observations, SCUFF-EM, an open-source software implementation of the boundary-element method, was employed in the calculation. The gold nanorod was modeled as a cylinder with two hemispherical tips at both ends. The diameter and length of the gold nanorod are 24 and 69 nm respectively, and the gap was set at 1 nm. Linearly polarized waves with the polarizations parallel (S) or perpendicular (P) to the symmetric plane of the dimer was used as excitation source in the calculations. The dielectric constant of bulk gold obtained from literature values was used [32]. Figures 5a and b show the calculated scattering cross sections for the dimer structures with various *θ* under the excitation of light with S- and P-polarizations, respectively. With the increase of the structure angle *θ*, the scattering intensity of the antibonding mode gradually decreases while that of the bonding mode gradually increases, which is consistent with the experimental observations. The peak wavelengths of the two resonance modes, however, were almost unchanged at fixed values (Fig. 5c). This is because the hybridized energy is mainly dependent on the coupling strength that is determined by the gap distance [35]. Considering the dimer structures with varying structure angles possess the same gap distance, it is reasonable to find the wavelengths of the two resonance modes independent of the structure angle. We further investigate the refractive index sensitivity of the dimer structures with various structure angles. The refractive index of the surrounding medium was set as *n* = 1.28, 1.30, 1.32 in the index sensitivity calculations. With it evaluated and plotted as a function of *θ* (Fig. 5d), we find the sensitivity is independent of the structure angle.

## Conclusions

In conclusion, we performed a systematic study on the angle-resolved plasmonic properties of single gold nanorod dimer structures both experimentally and theoretically. Strong plasmonic coupling occurs in these dimer structures, which results in the hybridized bonding and antibonding resonance modes. Single-particle dark-field scattering experiments indicate that the modes both are highly sensitive to the angle between the excitation polarization and the orientation of the dimer. The scattering intensity follows a typical cos^2^*φ* dependence due to the decomposition of the excitation electric field vector along either the bisector of the dimer for the radiative antibonding mode or its orthogonal direction for the radiative bonding mode. Effect of the angle between the two nanorods in the dimer on the scattering is also investigated by combining SEM and single-particle scattering spectroscopy. Both analytical and numerical methods are used in the calculations. The resonance wavelengths as well as the refractive index sensitivities are found independent of the structure angle. The calculated results are in good agreement with the measurements. Knowledge gained is helpful for the design of more complicated plasmonic structures, such as Fano and chiral structures. It is of particular interest for applications because these dimer structures can be used as building blocks in plasmon-based optical and optoelectronic devices.

## Electronic supplementary material

Below is the link to the electronic supplementary material.

Supplementary material 1 (PDF 224 kb)
