# Supplementary material for: Angle-Resolved Plasmonic Properties of Single Gold Nanorod Dimers
Source: Nanomicro Lett. 2014 Sep 26;6:372–80. doi: 10.1007/s40820-014-0011-7 (PMC6223963; doi:10.1007/s40820-014-0011-7)
Supplement: Supplementary file 1 — Supplementary material 1 (PDF 224 kb) [file 40820_2014_11_MOESM1_ESM.pdf]

Supporting Information for

Angle-resolved Plasmonic Properties of Single Gold Nanorod Dimers

Jian Wu[1,2], Xuxing Lu[2], Qiannan Zhu[2], Junwei Zhao[2], Qishun Shen[1], Li Zhan[1,*], Weihai Ni[2,*]

[1]Department of Physics and Astronomy, Key Laboratory for Laser Plasmas (Ministry of Education), State Key Lab of Advanced Optical Communication Systems and Networks, Shanghai Jiao Tong University, Shanghai, 200240, P. R. China

[2]Division of *i*-Lab, Key Laboratory for Nano-Bio Interface Research & Collaborative Innovation Center of Suzhou Nano Science and Technology, Suzhou Institute of Nano-Tech & Nano-Bionics, Chinese Academy of Sciences, Suzhou, Jiangsu, 215123, P. R. China

*Corresponding authors. E-mail: whni2012@sinano.ac.cn, lizhan@sjtu.edu.cn

Experimental Section

1 Chemicals

All the chemicals were commercially obtained. Tetrachloroauric acid ($HAuCl_4 \times 3H_2O$) was purchased from Acros. Odium borohydride ($NaBH_4$) and cetyltrimethylammonium bromide (CTAB), hydrochloric acid 36 % (HCl) were supplied by Sinopharm. L-ascorbic acid (AA), silver nitrate (AgNO3), trisodium citrate and L- cysteine (CYS) were bought from Aldrich. Milli-Q water with a resistivity higher than 18.2 MΩ/cm was used in all preparations.

2 Preparation of Au Nanorods

The Au nanorods were prepared using Ag ion-assisted seed mediated method [1-4] . The seed solution for Au nanorods growth was prepared as follows: 5 mL of 0.5 mM $HAuCl_4$ was mixed with 5 mL of 0.2 M CTAB solution in a 20 mL scintillation vial. 0.6 mL of fresh 0.01 M NaBH4 was diluted to 1 mL with water and was then injected to the Au(III)-CTAB solution under vigorous stirring (1200 rpm). The solution color changed from yellow to brownish yellow and the stirring was stopped after 2 min. The seed solution was aged at 30°C for 30 min before use [3, 4]. The growth solution was made by first mixing together aqueous solutions of $HAuCl_4$ (0.01 M, 0.5 mL), $AgNO_3$ (0.01 M, 0.1 mL), and an aqueous CTAB (0.1 M, 10 mL) solution. An aqueous HCl solution (1.0 M, 0.2 mL) was then added, followed by the addition of freshly prepared aqueous ascorbic acid solution (0.1 M, 0.08 mL). After the resultant solution was mixed by inversion, 3.75 μL of the seed solution was added. The reaction mixture was subjected to gentle inversion for 2 min and then left undisturbed at room temperature over night. The silver nitrate and excess ascorbic acid were removed through one washing cycles consisting of centrifugation (30 min at 5000 RPM, Eppendorf 5800) followed by replacement of the supernatant with

approximately 10 mL of 0.1M CTAB. The Au nanorods dimensions measured from TEM are 69.3 ± 4.9 × 23.6 ± 1.8 nm, with an aspect ratio of 2.9 ± 0.3. See Fig. S1 for extinction spectrum and TEM image. Au nanorods were centrifuged after preparation to remove CTAB. Final concentration about 10 nM [5].

3 Assembly of Au Nanorods

The formation of the Au nanorods dimers was realized by using L-cysteine as linking molecular, according to the procedure described previously [6-11]. For the assembly, 0.3 mL of the stock Au nanorods solution was diluted to 1 mL with water, the pH of the solution was adjusted through adding HCl solutions (0.03 mL, 1 M) and followed by inversion for several times. 0.03 mL volume of CYS (30 mM) aqueous solution was then added to the gold nanorod solution to reach the desired concentrations. The mixture solution sample was transferred into a cuvette and placed in the thermal static cell holder of the spectrophotometer. The assembly process in the mixture solvent was monitored by recording the time-dependent extinction spectra [7, 12].

The assemblies were deposited on carbon-coated grid and cleaned cover glass (Deckglaser, Microscope Cover Glass) for TEM/SEM characterization and dark-field imaging. The two nanorods in each dimer are connected at the ends and form different angles. For the deposition of dimer onto the functionalized substrates, 0.07 mL of assembly solution dripped on the substrates, rinsed 5 mins later with ethanol, and dry without disturbed.

4 Characterizations

Extinction spectra of Au nanorod solutions were taken using a plastic cuvette of 1-cm path length on an Cary 60 UV/visible spectrophotometer. SEM imaging of cover glass specimens was carried out on an FEI Quanta 250 FEG microscope. TEM and HRTEM imaging were performed on an FEI Tecnai 20 Transmission microscope (operating at 200 kV).

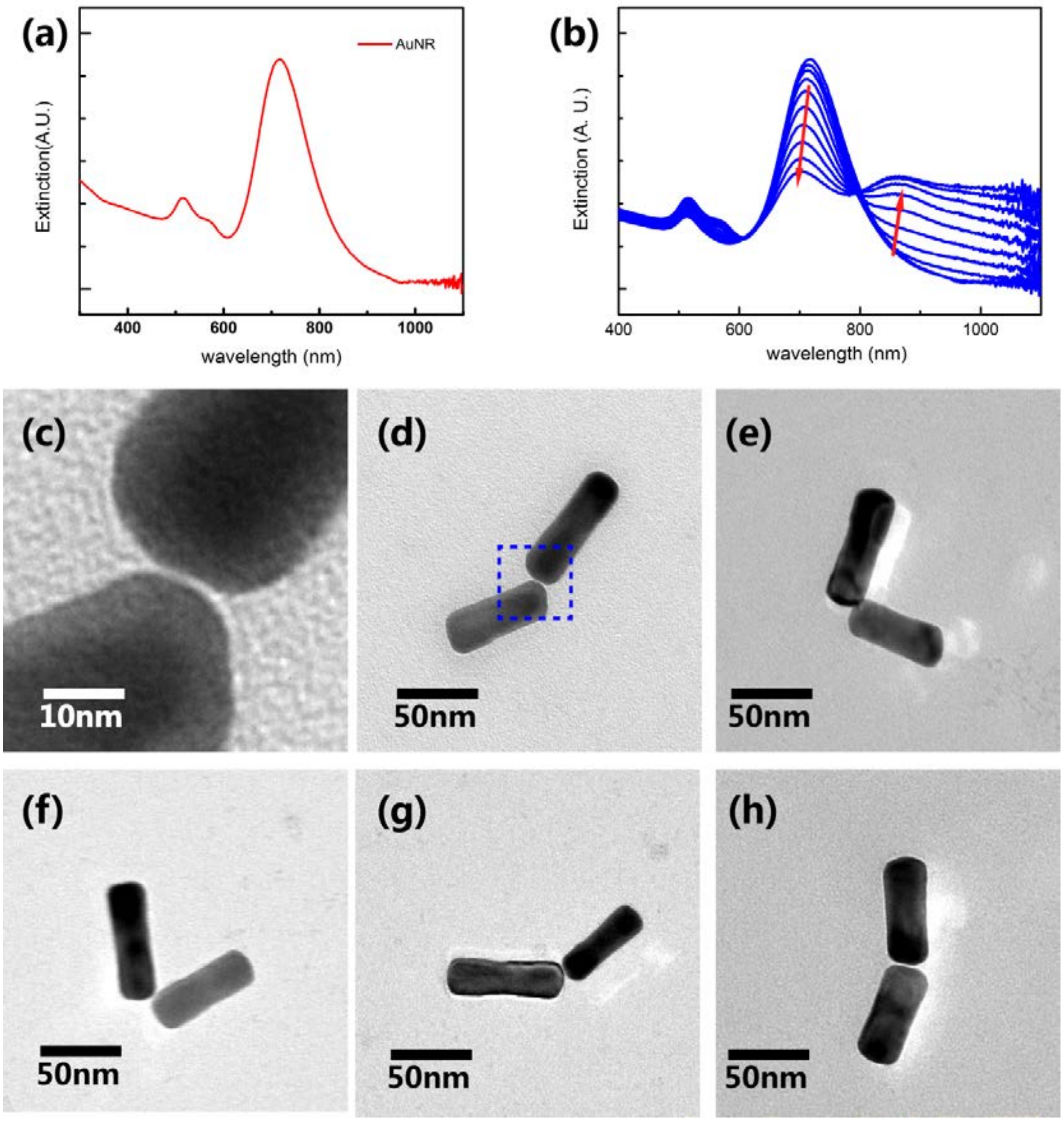

Fig. S1 (a) Extinction spectrum of as-synthesized Au nanorods in aqueous solution. The longitudinal plasmon resonance wavelength of the resulting nanorods is 717 nm. (b) Extinction spectra of CYS-assisted assembly recorded after the addition of the CYS. The spectra were recorded for 100 min. The solution sample was placed in a bath of 40°C. The interval between two consecutive spectra was 10 min. (d-e) Representative TEM images of assembled Au nanorod dimer, revealing the strong tendency toward end-to-end assembly. The angles between the two nanorods are 142°, 94°, 64°, 138° and 151°, respectively. (c) High resolution TEM image of the area indicated with a dashed box in (d), showing the presence of a gap between the two nanorods.

References

[1] B. Nikoobakht, M.A. El-Sayed, Preparation and growth mechanism of gold nanorods (NRs) using seed-mediated growth method. Chem. Mater. **15**(10), 1957-1962 (2003). doi:10.1021/cm020732l

[2] A. Gole, C.J. Murphy, Seed-mediated synthesis of gold nanorods: role of the size and nature of the seed. Chem. Mater. **16**(19), 3633-3640 (2004). doi:10.1021/cm0492336

[3] X. Ye, Y. Gao, J. Chen, D.C. Reifsnyder, C. Zheng, C.B. Murray, Seeded growth of monodisperse gold nanorods using bromide-free surfactant mixtures. Nano Lett. **13**(5), 2163-2171 (2013). doi:10.1021/nl400653s

[4] X. Ye, J.A. Millan, M. Engel, J. Chen, B.T. Diroll, S.C. Glotzer, C.B. Murray, Shape alloys of nanorods and nanospheres from self-assembly. Nano Lett. **13**(10), 4980-4988 (2013). doi:10.1021/nl403149u

[5] L.V. Brown, H. Sobhani, J.B. Lassiter, P. Nordlander, N.J. Halas, Heterodimers: plasmonic properties of mismatched nanoparticle pairs. ACS Nano. **4**(2), 819-832 (2010). doi:10.1021/nn9017312

[6] W. Ni, X. Kou, Z. Yang, J. Wang, Tailoring longitudinal surface plasmon wavelengths, scattering and absorption cross sections of gold nanorods. ACS Nano. **2**(4), 677-686 (2008). doi:10.1021/nn7003603

[7] W. Ni, R.A. Mosquera, J. Pérez-Juste, L.M. Liz-Marzán, Evidence for hydrogen-bonding-directed assembly of gold nanorods in aqueous solution. The J. Phys. Chem. Lett. **1**(8), 1181-1185 (2010). doi:10.1021/jz1002154

[8] P.K. Sudeep, S.T.S. Joseph, K.G. Thomas, Selective detection of cysteine and glutathione using gold nanorods. J. Am. Chem. Soc. **127**(18), 6516-6517 (2005). doi:10.1021/ja051145e

[9] X. Kou, S. Zhang, Z. Yang, C.-K. Tsung, G.D. Stucky, L. Sun, J. Wang, C. Yan, Glutathione- and cysteine-induced transverse overgrowth on gold nanorods. J. Am. Chem. Soc. **129**(20), 6402-6404 (2007). doi:10.1021/ja0710508

[10] P. Pramod, K.G. Thomas, Plasmon coupling in dimers of Au nanorods. Adv. Mater. **20**(22), 4300-4305 (2008). doi:10.1002/adma.200703057

[11] J. Kumar, X. Wei, S. Barrow, A.M. Funston, K.G. Thomas, P. Mulvaney, Surface plasmon coupling in end-to-end linked gold nanorod dimers and trimers. Phys. Chem. Chem. Phys. **15**(12), 4258-4264 (2013). doi:10.1039/C3CP44657C

[12] L. Shao, K.C. Woo, H. Chen, Z. Jin, J. Wang, H.-Q. Lin, Angle- and energy-resolved plasmon coupling in gold nanorod dimers. ACS Nano 4(6), 3053-3062 (2010). doi:10.1021/nn100180d
